# Identification and Validation of an Individualized Prognostic Signature of Bladder Cancer Based on Seven Immune Related Genes

**DOI:** 10.3389/fgene.2020.00012

**Published:** 2020-02-05

**Authors:** Huaide Qiu, Xiaorong Hu, Chuan He, Binbin Yu, Yongqiang Li, Jianan Li

**Affiliations:** ^1^Center of Rehabilitation Medicine, The First Affiliated Hospital of Nanjing Medical University, Nanjing, China; ^2^School of Rehabilitation Medicine, Nanjing Medical University, Nanjing, China; ^3^Department of Rehabilitation Medicine, The Affiliated Jiangsu Shengze Hospital of Nanjing Medical University, Nanjing, China

**Keywords:** immune related genes, prognostic classifier, bladder cancer, signature, nomogram

## Abstract

**Background:**

There has been no report of prognostic signature based on immune-related genes (IRGs). This study aimed to develop an IRG-based prognostic signature that could stratify patients with bladder cancer (BLCA).

**Methods:**

RNA-seq data along with clinical information on BLCA were retrieved from the Cancer Genome Atlas (TCGA) and gene expression omnibus (GEO). Based on TCGA dataset, differentially expressed IRGs were identified *via* Wilcoxon test. Among these genes, prognostic IRGs were identified using univariate Cox regression analysis. Subsequently, we split TCGA dataset into the training (n = 284) and test datasets (n = 119). Based on the training dataset, we built a least absolute shrinkage and selection operator (LASSO) penalized Cox proportional hazards regression model with multiple prognostic IRGs. It was validated in the training dataset, test dataset, and external dataset GSE13507 (n = 165). Additionally, we accessed the six types of tumor-infiltrating immune cells from Tumor Immune Estimation Resource (TIMER) website and analyzed the difference between risk groups. Further, we constructed and validated a nomogram to tailor treatment for patients with BLCA.

**Results:**

A set of 47 prognostic IRGs was identified. LASSO regression and identified seven BLCA-specific prognostic IRGs, i.e., RBP7, PDGFRA, AHNAK, OAS1, RAC3, EDNRA, and SH3BP2. We developed an IRG-based prognostic signature that stratify BLCA patients into two subgroups with statistically different survival outcomes [hazard ratio (HR) = 10, 95% confidence interval (CI) = 5.6–19, P < 0.001]. The ROC curve analysis showed acceptable discrimination with AUCs of 0.711, 0.754, and 0.772 at 1-, 3-, and 5-year follow-up respectively. The predictive performance was validated in the train set, test set, and external dataset GSE13507. Besides, the increased infiltration of CD4^+^ T cells, CD8+ T cells, macrophage, neutrophil, and dendritic cells in the high-risk group (as defined by the signature) indicated chronic inflammation may reduce the survival chances of BLCA patients. The nomogram demonstrated to be clinically-relevant and effective with accurate prediction and positive net benefit.

**Conclusion:**

The present immune-related signature can effectively classify BLCA patients into high-risk and low-risk groups in terms of survival rate, which may help select high-risk BLCA patients for more intensive treatment.

## Introduction

Bladder cancer (BLCA) is the most common malignancy of the urinary system with high morbidity and mortality rates (Bray et al., 2018). Approximately 25% of BLCA patients are diagnosed with muscle-invasive or metastatic disease during the early stages of prognosis (Akbani et al., 2017). Meanwhile patients with non-muscle-invasive BLCA continue to suffer from the high progression rates (Cambier et al., 2016; Chen et al., 2019). Overall, the 5-year survival rate at all stages of bladder cancer remains no more than 20% (Aggen and Drake, 2017). Once the tumor progresses to locally advanced or metastatic stage, standard treatment for BLCA with combination chemotherapy are insufficient with low response and survival rates (Von et al., 2000; Maase et al., 2006). With the emergence of immune checkpoint therapy including programmed cell death protein (PD-1) and programmed death-ligand 1 (PD-L1), the treatment of advanced BLCA patients with durable response has become possible (Koshkin and Grivas, 2018). However, most BLCA patients do not adequately respond to PD-1 or PD-L1-targeted therapy; and hence, it is imperative to develop prognostic biomarkers to closely monitor progression and shed light on treatment stratification.

The most representative of BLCA type is urothelial cancer (UC). Up to 30% of urothelial cancer (UC) specimens have demonstrated differential expression in PD-L1, which is associated with increased all-cause mortality (Inman et al., 2007; Boorjian et al., 2008; Faraj et al., 2015). However, standalone PD-L1 expression acts as an unviable biomarker since significant heterogeneity of association were observed between PD-L1 staining and clinical results of BLCA patients (Rosenberg et al., 2016; Massard et al., 2017; Powles et al., 2017; Sharma et al., 2017; Bellmunt et al., 2017). Since PD-L1 expression is subject to immunohistochemistry (IHC) score; the expression of immune-related gene (IRG) may serve as a better biomarker as it can be quantified from multiple cell types within a sample (Sweis et al., 2016). In this context, the IRG-based prognostic signatures have been proposed for patients diagnosed with nonsquamous non–small cell lung cancer (Li et al., 2017) and papillary thyroid cancer (Lin et al., 2019), which show significant prognostic values. However, the clinical relevance and prognostic significance of IRGs-based signature in BLCA remains unknown.

The purpose of the present study was to investigate the clinical implications of IRGs on prognostic stratification and their potential as biomarkers for targeted BLCA therapy. In the manuscript, we performed integrated analysis using IRG expression profiles and clinical information of patients with BLCA retrospectively. Individualized prognostic signature based on IRGs was developed and validated in independent datasets, while underlying mechanisms were explored using bioinformatics analysis.

## Materials and Methods

### Data Source

From The Cancer Genome Atlas (TCGA), normalized RNA sequencing (RNA-Seq) data sets with estimation of Fragments Per Kilobase of transcript per Million mapped reads (FPKM) from 414 tumor samples and 19 non-tumor samples were retrieved. Clinical data were also derived from TCGA into integrated analysis. Gene expression data and clinical information in GSE13507 based on the Illumina human-6 v2.0 expression BeadChip platform were downloaded from gene expression omnibus (GEO) database (https://www.ncbi.nlm.nih.gov/geo/). A total of 568 patients were involved in the development and validation of the prognostic signature, i.e., 403 patients in TCGA dataset and 165 patients in GSE13507 cohort.

### Data Preprocessing and Differential Analysis

In the R software, differentially expressed genes (DEGs) between BLCA and normal tissues were identified using Wilcoxon test after within-array replicate probes were replaced with their average *via limma* package (Ritchie et al., 2015; Yue et al., 2019). The p-value was adjusted with the false discovery rate (FDR) (Benjamini and Hochberg, 1995). FDR < 0.05 and |log2(FC)| value > 1 was considered significant.

The Kyoto Encyclopedia of Genes and Genomes (KEGG) (Kanehisa and Goto, 2000) pathway enrichment were analyzed with the DEGs using the *clusterProfiler* R package (Yu et al., 2012). P < 0.05 was considered statistically significant.

### Development and Validation of a Prognostic Signature

By accessing the Immunology Database and Analysis Portal (IMMPORT) (Bhattacharya et al., 2014) website (https://www.immport.org), we retrieved a latest list of immune‐related genes, out of which we identified BLCA-specific immune‐related genes (IRGs) after matching the DEGs. Survival-associated IRGs were identified using univariate Cox regression analysis with a threshold value of p < 0.01.

Patients in TCGA dataset was randomly assigned in a 7:3 ratio to a training set and test set with the same proportion of each BLCA stage. With expression profiles of the identified survival-associated IRGs, we conducted least absolute shrinkage and selection operator (LASSO) regression analysis in the training set. Subsequently we calculated the individualized risk score with coefficients and constructed a prognostic signature which separates the high-risk BLCA patients from the low-risk group. Clinical relevance was validated using survival analysis between groups with thresholds of p < 0.05 using the R software survival and survminer package; whereas, the receiver operating characteristic (ROC) analysis was performed (*via* the survival ROC package), and the area under the curve (AUC) was calculated at multiple time-point to evaluate the discrimination (Heagerty et al., 2000).

Clinical characteristics including age, gender, stage, and tumor-node-metastasis (TNM) status were collected from TCGA database and integrated with transcriptome profile derived from TCGA dataset. Multivariate cox regression analysis was performed using clinical data and risk scores to see if the prognostic value of risk scores was independent of clinical characteristics. A value of p < 0.05 was considered significant statistically.

### External Validation of the Prognostic Signature in the Test Set and GSE13507 Cohort

The prognostic signature with the same risk score formula and cutoff value was then validated in the test set and external dataset GSE13507 respectively. We chose the GSE13507 as it involved the largest sample size of BLCA patients with survival data. Likewise, the prognostic model was presented as a risk plot in each dataset that encompassed the expression level of the included genes, distribution of risk score, and survival status of individuals.

### Gene Set Enrichment Analysis Analysis

We performed *Gene Set Enrichment Analysis* (GSEA) between high-risk and low-risk group as separated by the 7-IRG signature *via clusterProfiler* and *enrichplot* R package. Two functions (gseGO and gseKEGG) were applied to identify the enriched terms in Gene Ontology (GO) and KEGG with a false discovery rate (FDR) value < 0.05 (Subramanian et al., 2005).

### Difference of Tumor-Infiltrating Immune Cells Between Groups Defined by the Signature in Bladder Cancer

Six types of tumor-infiltrating immune cells were retrieved from Tumor Immune Estimation Resource (TIMER) (https://cistrome.shinyapps.io/timer/), (Li et al., 2017) also known as a web server for comprehensive analysis of tumor-infiltrating immune cells. The abundance of immune cells was tested one by one to detect the differences between the prognostic classified risk groups using Wilcoxon test.

### Construction of a Nomogram Based on the Immune-Related Gene Signature

A nomogram encompassing the risk score based on expression of prognostic IRGs and clinicopathological factors was constructed using the *rms* R package. Discrimination of the nomogram was validated using ROC analysis at 3- and 5-year follow-up, and predictive accuracy was tested by presenting the difference between predicted survival and actual survival using calibration plot. Further, decision curve analysis (DCA) was performed to examine the clinical utility of the nomogram by quantifying the net benefits at different threshold probabilities.

## Results

### Bladder Cancer Specific Immune-Related Genes

**Figure 1:**
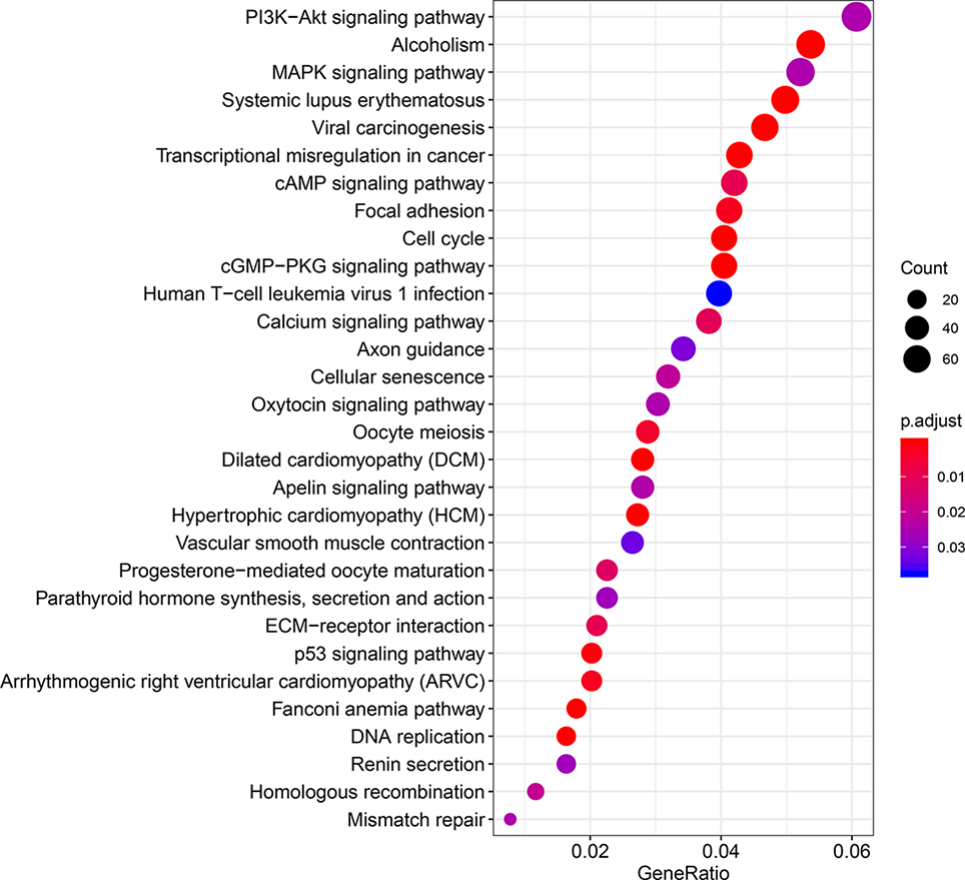
The top 30 most enriched Kyoto Encyclopedia of Genes and Genomes pathways.

**Figure 2:**
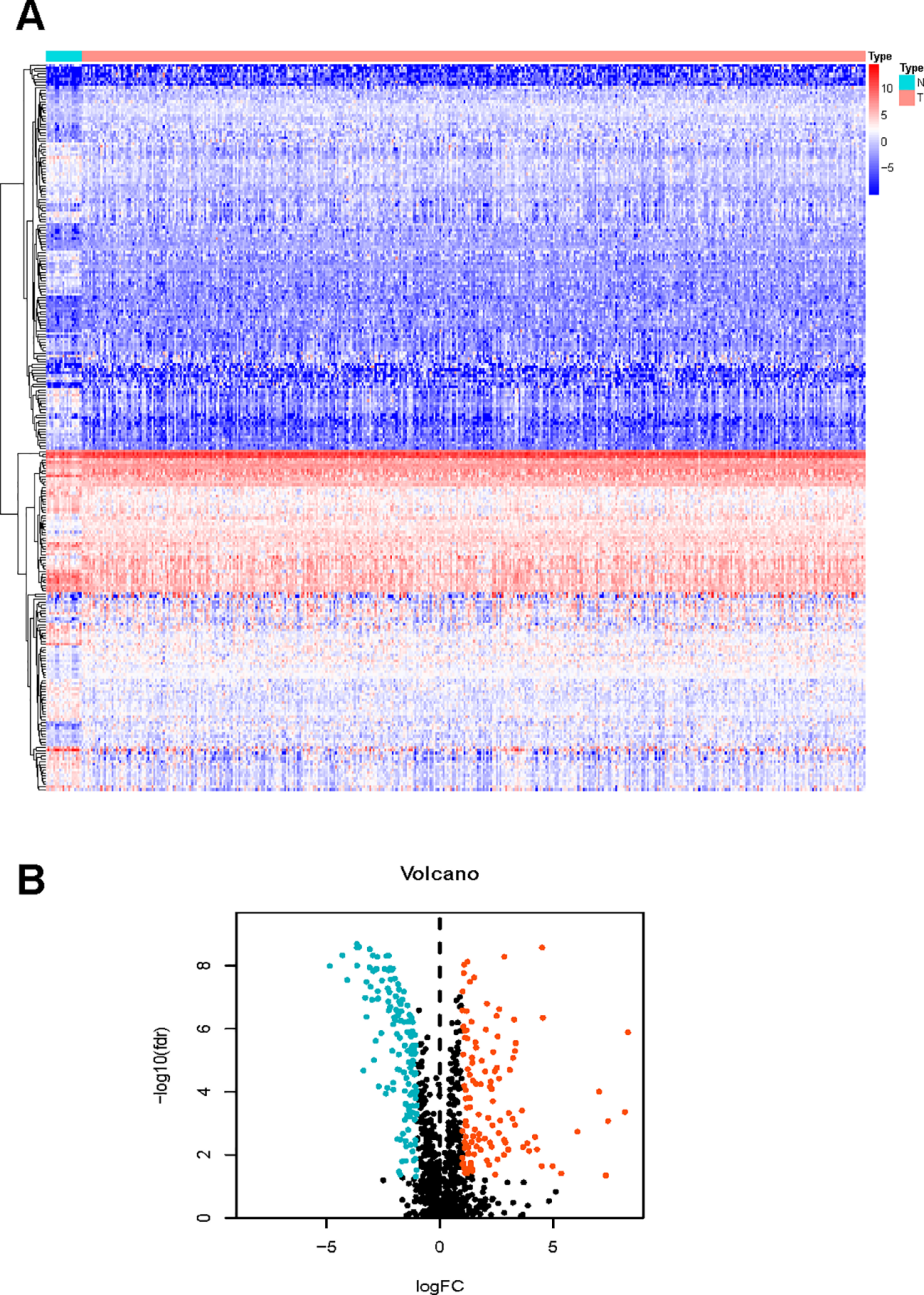
Differentially expressed immune‐related genes. Heatmap **(A)** and a volcano plot **(B)**.

We managed to obtain 4,876 DEGs base on the transcription profile in TCGA dataset, out of which 3,453 genes were upregulated and 1,423 downregulated. The DEGs were presented in **Supplementary Material S1**. The KEGG analysis (**Figure 1**) indicated that the genes were mainly involved in PI3K−Akt and MAPK signaling pathway, which are pivotal in the regulation of immune responses (Liu et al., 2007; Weichhart and Säemann, 2008). Specific IRGs were identified with intersection of the immune-related genes and DEGs in BLCA. As shown in **Figure 2**, we identified 120 upregulated, and 140 downregulated BLCA-specific IRGs.

### Development and Internal Validation of a Prognostic Signature

**Figure 3:**
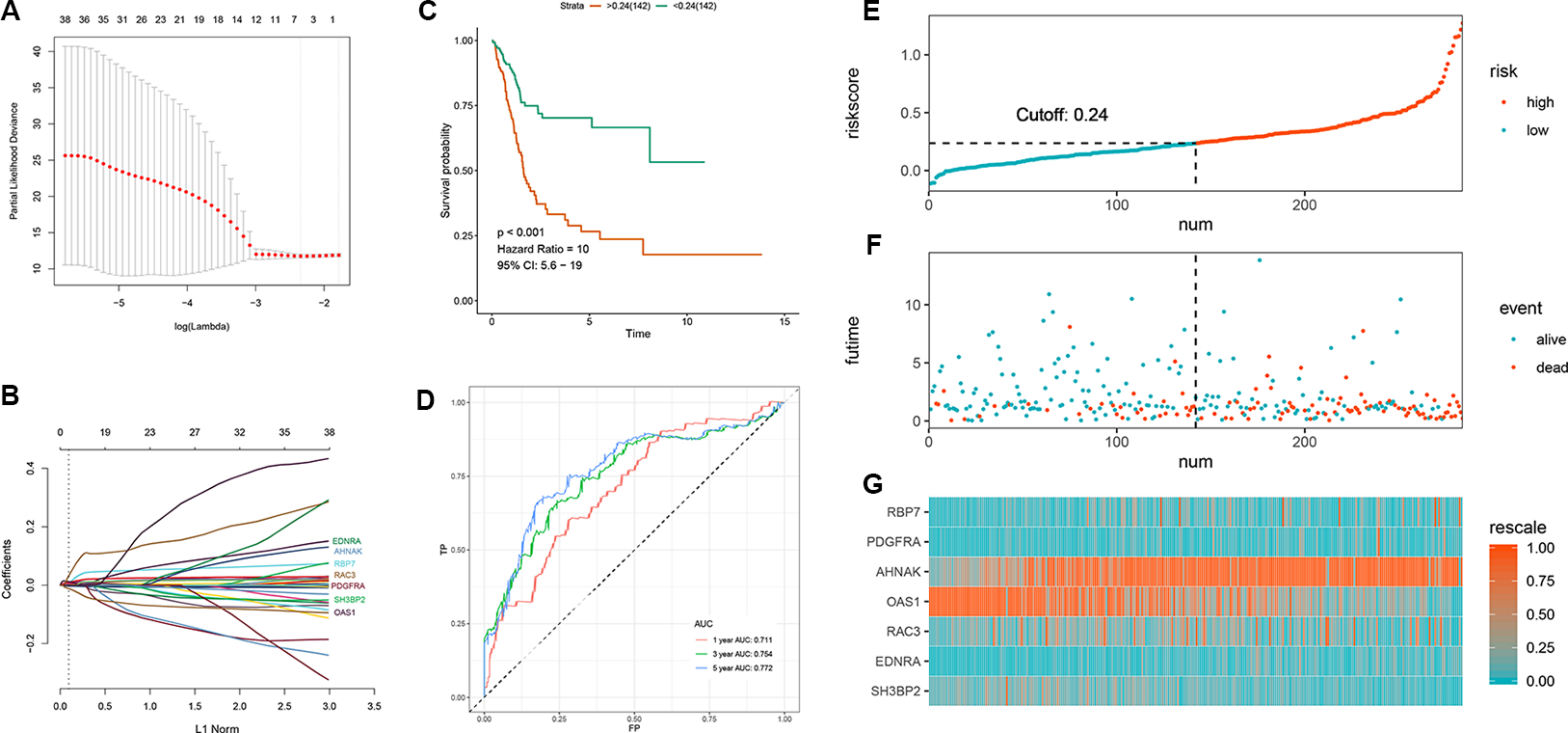
Development of the prognostic signature based on seven immune‐related genes (IRGs) in the training set. **(A, B)** LASSO regression identified 7 IRGs **(C)** Time-dependent ROC curve of the 7-IRG prognostic signature **(D)** Survival analysis between signature-defined risk groups **(E)** Heatmap of expression profiles of included IRGs **(F)** Distribution of groups based on the signature **(G)** Survival status of patients in different groups.

A total of 403 patients with BLCA in TCGA dataset was recorded with follow-up time >0. Based on the data in these patients, totally 47 survival-associated IRGs were identified using univariate Cox regression. **Table 1** shows the general profile of survival-associated IRGs in BLCA. Subsequently, we split TCGA dataset randomly into training set (n = 284) and test set (n = 119). With expression profiles of survival-associated IRGs, we conducted a LASSO regression in the training set and identified seven BLCA-specific prognostic IRGs (**Figures 3A, B**). The boxplot of expression levels of these genes between BLCA and normal samples in TCGA dataset along with results of Wilcoxon test is provided in **Supplementary Material S2**.

**Table 1:** Characteristics of bladder cancer‐specific immune‐related genes (univariate cox analysis).

id	HR	HR.95L	HR.95H	p value
PTGER3	1.3982	1.2271	1.5932	0.0000
TACR1	1.5641	1.2256	1.9961	0.0003
IGF1	1.3292	1.1747	1.5039	0.0000
GHR	1.2524	1.0956	1.4317	0.0010
SLIT2	1.1783	1.0856	1.2790	0.0001
NR3C2	1.1619	1.0673	1.2648	0.0005
GLP2R	1.1511	1.0672	1.2416	0.0003
AGTR1	1.1437	1.0661	1.2270	0.0002
FGF10	1.1685	1.0575	1.2911	0.0022
SEMA3E	1.2198	1.0529	1.4131	0.0081
NGF	1.1376	1.0521	1.2300	0.0012
EDNRA	1.0914	1.0496	1.1349	0.0000
ADIPOQ	1.0894	1.0438	1.1369	0.0001
SEMA3A	1.1546	1.0428	1.2785	0.0057
PDGFD	1.0752	1.0370	1.1149	0.0001
NFATC1	1.1038	1.0299	1.1830	0.0052
AKT3	1.0724	1.0284	1.1183	0.0011
IL17RD	1.0712	1.0245	1.1201	0.0025
PDGFRA	1.0444	1.0213	1.0680	0.0001
ILK	1.0637	1.0204	1.1088	0.0036
NFATC4	1.0614	1.0195	1.1050	0.0037
NRP2	1.0482	1.0189	1.0784	0.0011
KCNH2	1.0335	1.0188	1.0484	0.0000
IL34	1.0409	1.0156	1.0667	0.0014
ANGPTL1	1.0294	1.0133	1.0457	0.0003
OGN	1.0280	1.0131	1.0430	0.0002
RAC3	1.0239	1.0130	1.0350	0.0000
PAEP	1.0410	1.0124	1.0704	0.0047
PGF	1.0297	1.0118	1.0480	0.0011
S1PR1	1.0300	1.0084	1.0521	0.0063
PPY	1.0169	1.0078	1.0261	0.0003
AHNAK	1.0119	1.0077	1.0160	0.0000
TGFBR2	1.0173	1.0076	1.0271	0.0005
ELN	1.0183	1.0075	1.0292	0.0009
NAMPT	1.0135	1.0070	1.0201	0.0000
RBP7	1.0134	1.0069	1.0199	0.0001
CXCL12	1.0117	1.0060	1.0175	0.0001
ANXA6	1.0094	1.0040	1.0149	0.0006
PTX3	1.0095	1.0037	1.0153	0.0012
THBS1	1.0018	1.0007	1.0029	0.0017
CSRP1	1.0019	1.0005	1.0034	0.0089
TPM2	1.0010	1.0003	1.0016	0.0056
A2M	1.0012	1.0003	1.0021	0.0098
MMP9	1.0002	1.0001	1.0004	0.0093
TAP1	0.9950	0.9915	0.9986	0.0061
OAS1	0.9855	0.9769	0.9942	0.0011
SH3BP2	0.8812	0.8154	0.9523	0.0014

After extracting the coefficient values, we calculated individualized risk scores with coefficient‐weighted expression levels of seven IRGs with the following formula:

Risk score=expression level of RBP7∗0.0044+PDGFRA∗0.0021+AHNAK ∗0.0054+OAS1∗(−0.0021)+RAC3∗0.0043+EDNRA∗0.0142+SH3BP2∗(−0.0019)

The median of the risk score is 0.24. Individuals with a risk score higher than 0.24 were classified as a high-risk group while the other as a low-risk group; consequently, a prognostic signature based on seven IRGs was developed. The survival analysis indicated that the survival rate was remarkably lower in the high‐risk group as opposed to low‐risk group [hazard ratio (HR) = 10, 95% confidence interval (CI) = 5.6–19, p-value <0.001, **Figure 3C**]; whereas, the ROC curve analysis (**Figure 3D**) showed acceptable discrimination with AUCs of 0.711, 0.754, and 0.772 at 1-, 3-, and 5-year follow-up respectively. **Figures 3E–G** represents the risk plot encompassing distribution of groups based on the signature, survival status of individuals between groups, and the expression level of included IRGs. It shows a clear separation of survival status between risk groups with red dots being death and blue ones alive. While a large amount of death occurred in the high-risk group, most of the patients in low-risk group stayed alive at follow-up.

**Figure 4:**
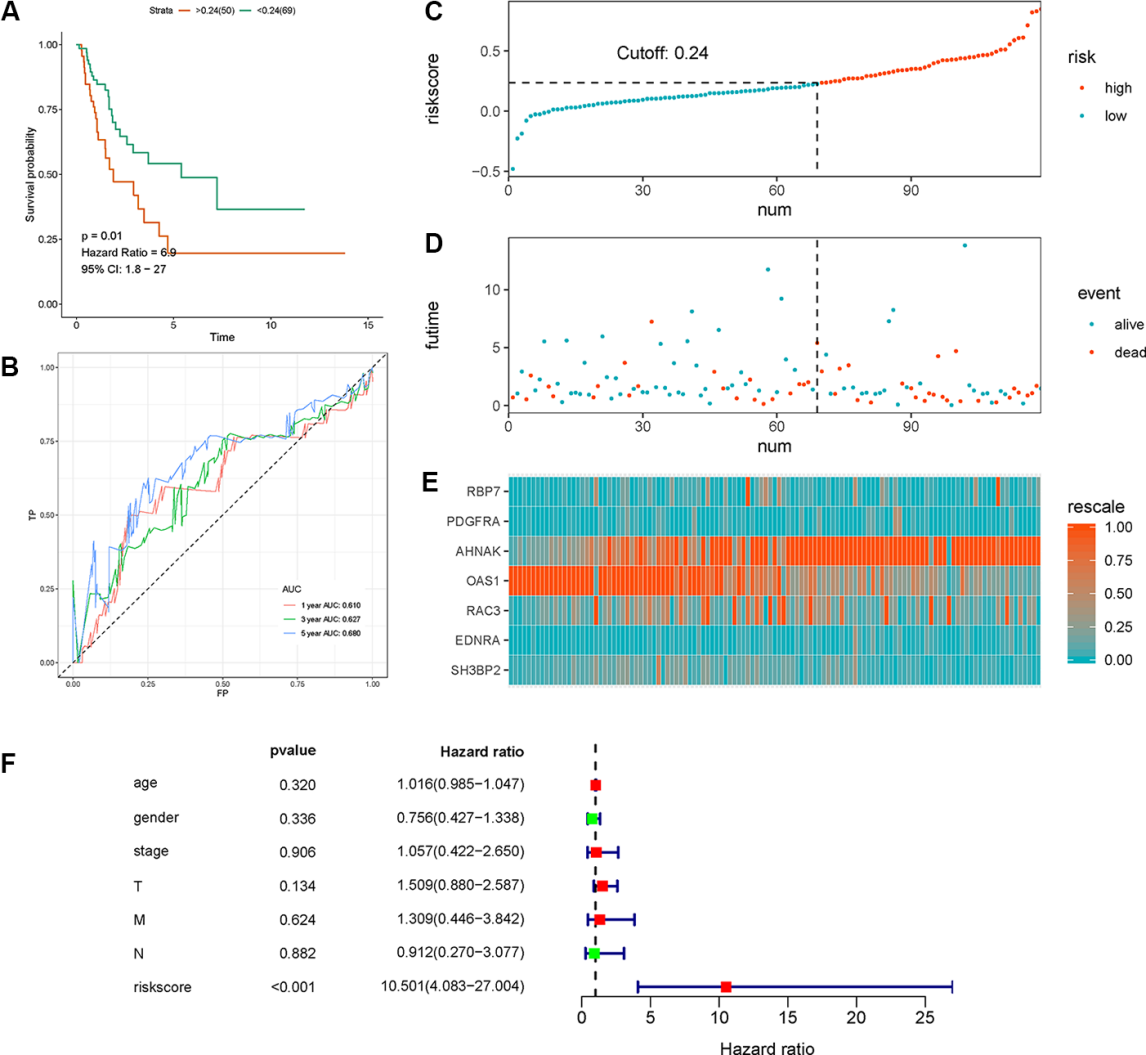
Validation of the the seven immune‐related gene (7-IRG) prognostic signature in the test set. **(A)** Time-dependent receiver operating characteristic (ROC) curve of the 7-IRG prognostic signature. **(B)** Survival analysis between signature-defined risk groups. **(C)** Heatmap of expression profiles of included IRGs. **(D)** Distribution of groups based on the signature. **(E)** Survival status of patients in different groups. **(F)** Multivariable analyses of the risk score, age, gender, tumor stage, and T/N/M, where confidence interval (CI) and hazard ratio (HR) stand for a confidence interval, and hazard ratio, respectively.

We calculated individual risk score with the aforementioned formula and classified the patients in the test set into high-risk and low-risk groups. Similarly, we validated the clinical utility and discrimination in both datasets. **Figure 4** summarizes the results of validation in the test set. A significant separation was shown in the Kaplan-Meier survival curve in the test set (HR = 6.9, 95% CI = 1.8–27, p-value = 0.01, **Figure 4A**). ROC curve analysis demonstrated acceptable discrimination with an AUC of 0.68 in predicting 5-year overall survival (**Figure 4B**). Meanwhile, the risk plot shows markedly different survival status between groups (**Figures 4C–E**).

Multivariate Cox regression analysis with TCGA dataset suggested that the 7-IRG based prognostic signature could be an independent predictor after other variables including age, gender, stage, and information coded in TNM concerning status of tumor, lymph node, and distant metastasis were adjusted (**Figure 4F**).

### External Validation in the Test and GSE13507 Datasets

**Figure 5:**
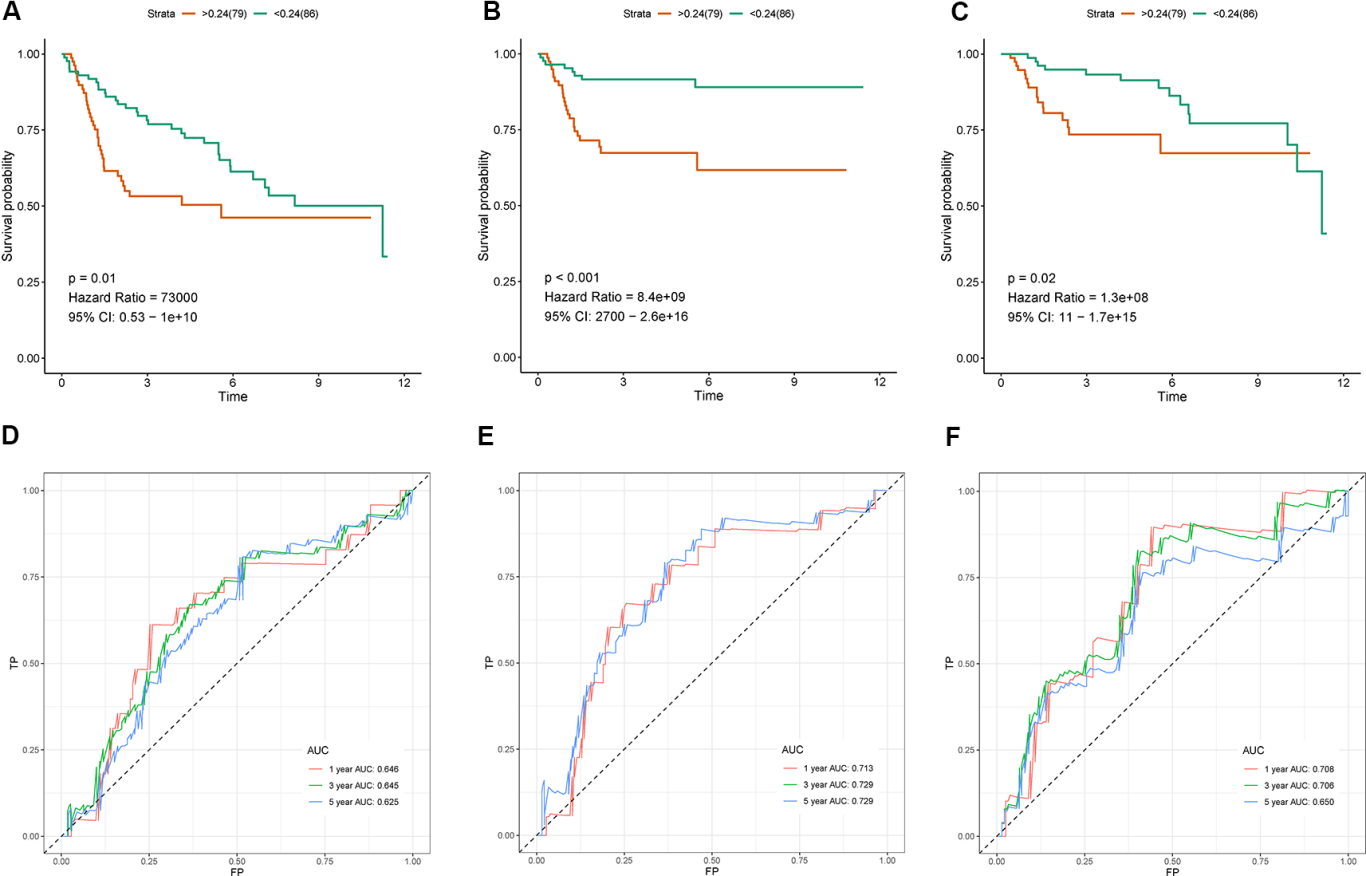
Validation of the the seven immune‐related gene (7-IRG) prognostic signature in GSE13507 cohort. **(A–C)** Survival analysis between signature-defined risk groups [overall survival (OS) **(A)**, cancer-specific survival (CSS) **(B)**, and progression-free survival (PFS) **(C)**]. **(D–F)** Time-dependent receiver operating characteristic (ROC) curve for predicting survival of the 7-IRG prognostic signature [OS **(D)**, CSS **(E)**, and PFS **(F)**].

In GSE13507 cohort, we validated the IRG-based prognostic signature for overall survival, cancer specific survival and progression-free survival using the same formula and cutoff value. The results were presented in **Figure 5** with Kaplan-Meier curve and time-dependent ROC curve. Consistent with above findings, significantly different survival outcomes were observed between risk groups in overall survival (OS) (HR = 73,000, 95% CI = 0.53–10^10, p-value = 0.01, **Figure 5A**), cancer-specific survival (CSS) (HR = 8.4*10^9, 95% CI = 2,700–2.6*10^16, p-value <0.001, **Figure 5B**), and progression-free survival (PFS) (HR = 1.3*10^8, 95% CI = 11-1.7*10^15, p-value = 0.02, **Figure 5C**). Similarly, ROC curve analysis indicated that the prognostic signature can effectively predict 65% of the overall survival, 71% of the cancer specific survival, and 71% of the progression-free survival at 1- and 3-year follow-up (**Figures 5D, E**). Risk plots for different survival indicators were presented in **Supplementary Material S3**.

### Gene Set Enrichment Analysis

**Figure 6:**
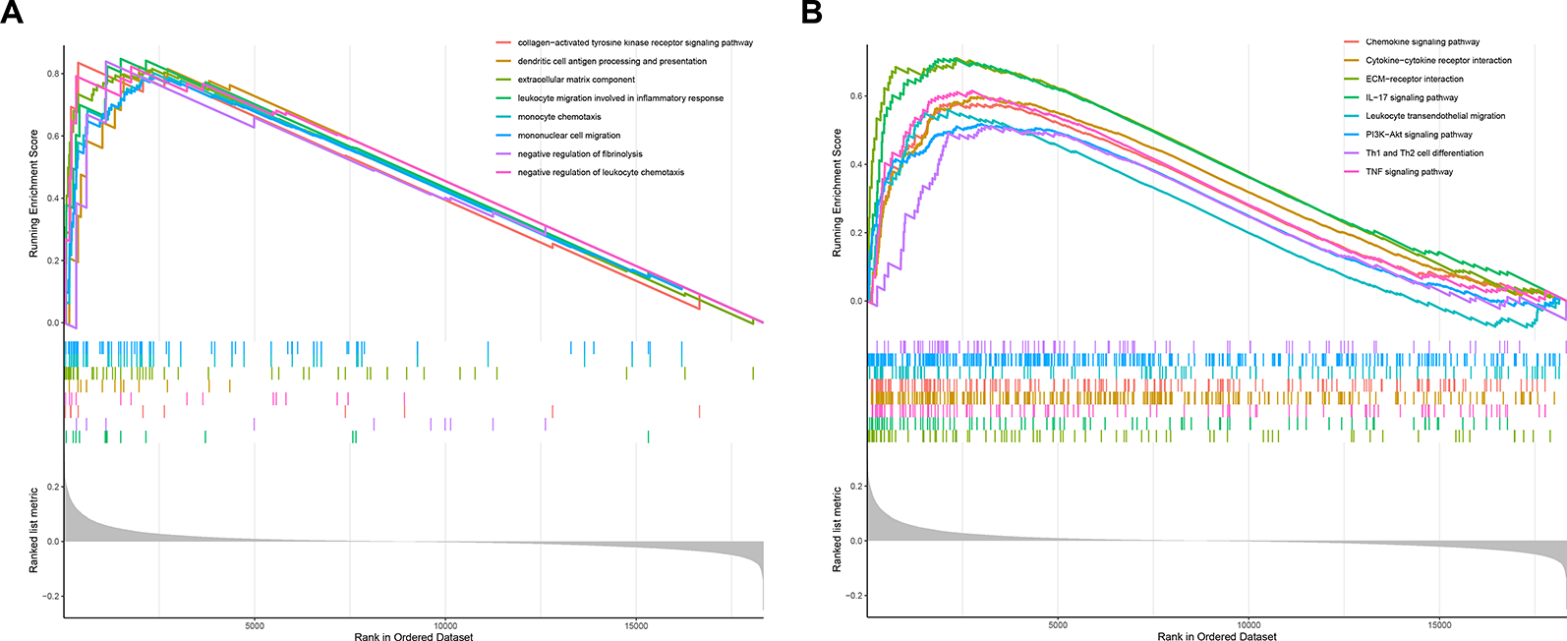
*Gene Set Enrichment Analysis* (GSEA) analysis between risk groups as classified by the immune-related gene (IRG)-based signature. **(A)** GSEA in gene ontology (GO) terms. **(B)** GSEA in Kyoto Encyclopedia of Genes and Genomes (KEGG) pathways.

The predictive performance of the 7-IRG signature could be associated with the biological function of these IRGs in BLCA. To explore the underlying mechanism, we performed GSEA between high-risk and low-risk groups based on the signature to identify the enriched GO term as well as KEGG pathway. **Figure 6** shows the results of enrichment analysis. GO terms (**Figure 6A**) including collagen−activated tyrosine kinase receptor signaling pathway, dendritic cell antigen processing and presentation, extracellular matrix component, as well as leukocyte migration and chemotaxis involved in inflammatory response were significantly enriched. In the case of KEGG pathway (**Figure 6B**), we found enhanced activity of several immune-related pathways in the high-risk group, such as chemokine signaling pathway, cytokine−cytokine receptor interaction, ECM−receptor interaction, IL−17 signaling pathway, leukocyte transendothelial migration, and PI3K−Akt signaling pathway. Particularly, upregulated PI3K−Akt pathway was in consistency with the KEGG enrichment of DEGs between tumor and control specimens in BLCA (**Figure 2**).

### Difference of Tumor-Infiltrating Immune Cells Between Risk Groups

**Figure 7:**
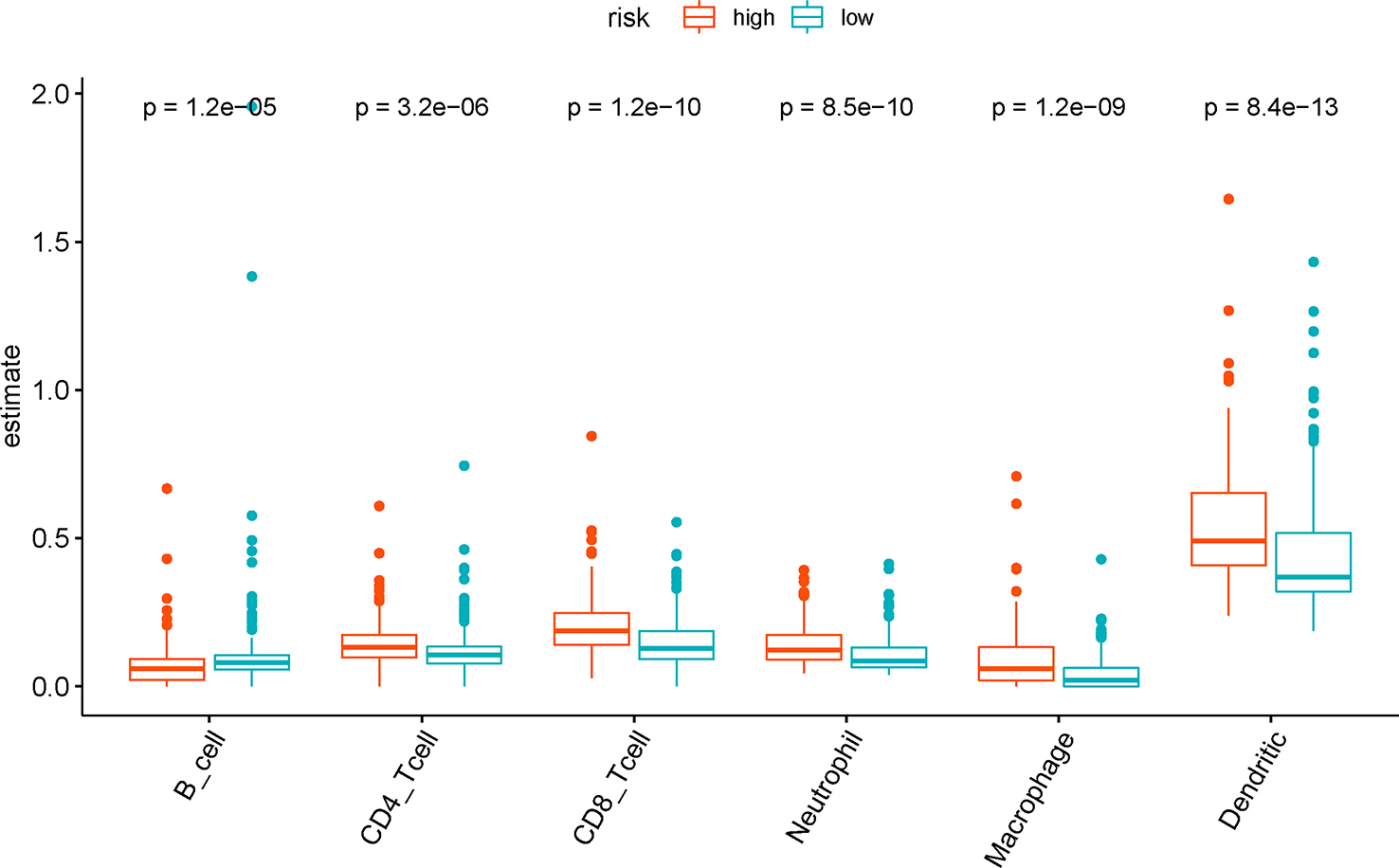
The difference of tumor-infiltrating immune cells among risk groups as defined by the seven immune‐related gene (7-IRG) prognostic signature.

We analyzed the difference in tumor-infiltrating immune cells in TCGA samples between the risk groups to explore the relationship between the present IRG-based prognostic signature and the tumor immune microenvironment. The results showed that abundance of six types of tumor-infiltrating immune cells except B cells were significantly enriched (P < 0.001) in the high risk group as compared to the low risk group by Wilcoxon test. By contrast, higher B cell level was observed in the low-risk group (P < 0.001) (**Figure 7**).

### Construction of a Nomogram Based on the Seven Immune-Related Gene Signature

**Figure 8:**
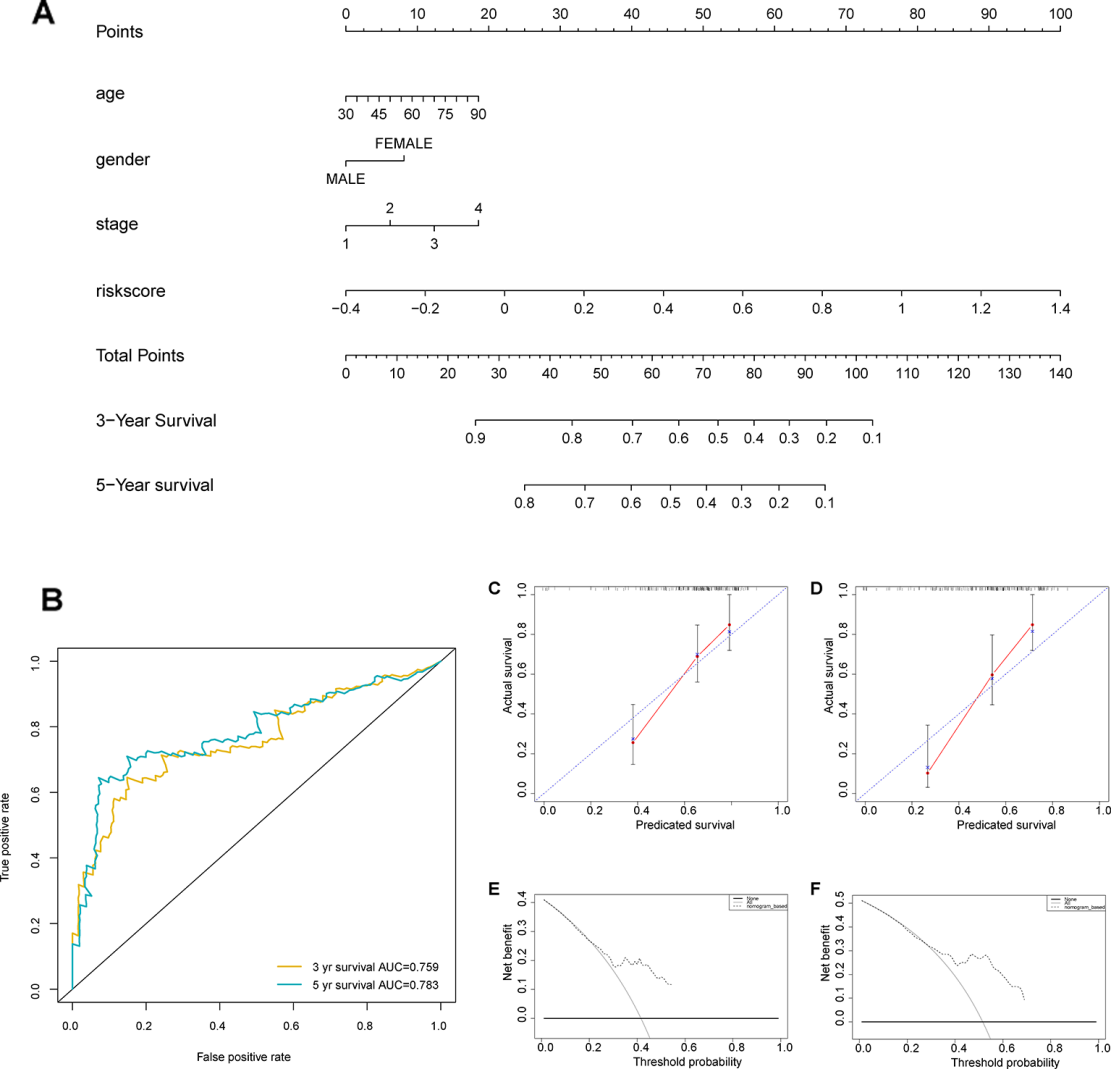
Construction of a nomogram based on the seven immune‐related gene (7-IRG) signature. **(A)** A nomogram based on the signature and clinical information. **(B)** Time-dependent receiver operating characteristic (ROC) curve for predicting overall survival (OS) of the nomogram. **(C, D)** Calibration plot evaluating the predictive accuracy of the nomogram [at 3-year survival **(C)** at 5-year survival **(D)**]. **(E, F)** Decision curve analysis evaluating the clinical utility of the nomogram [at 3-year survival **(E)** at 5-year survival **(F)**].

We developed a nomogram to predict 1-, 3-, and 5-year overall survival using the 7-IRG signature and the aforementioned clinical factors of colorectal cancer (**Figure 8A**). The ROC analysis (**Figure 8B**) showed adequate discrimination with an AUC of 0.759, and 0.783 at 3- and 5-year follow-up, indicating that the nomogram could distinguish over 75% of survival outcome at these time-points. In addition, the calibration plot (**Figure 8C-D**) demonstrated optimal predictive accuracy with predicted survival rate approximately equivalent to actual survival. Further, results of DCA (**Figure 8E-F**) showed that most of the dashed curve were above the two solid lines (black and gray), suggesting positive net benefits. In other words, clinical decision made upon the nomogram would be favorable than treat-none or treat-all scheme.

### Discussion

BLCA patients are at substantial risk for recurrence and metastasis. Over the past several decades, treatments for metastatic BLCA remained unsatisfied with platinum chemotherapy until immune checkpoint therapy came up (Bellmunt et al., 2014; Milowsky et al., 2016; Ghasemzadeh et al., 2016). With the growing popularity of immunotherapy, attention has been shifting to developing novel biomarkers related to tumor immune milieus for estimates of treatment response and survival outcome (Aggen and Drake, 2017).

Till date, no report on IRG-based prognostic signatures exists, although considerable efforts have been made to develop prognostic signatures based on differentially expressed genes (Gao et al., 2019; Yan et al., 2019; Zhang et al., 2019). We identified seven prognostic IRGs based on a comprehensive analysis which could serve as valuable biomarkers in the clinical setting. Further, the prognostic signature based on the seven IRGs could categorize BLCA patients into two subgroups with statistically different survival outcomes, which was validated in both TCGA and GSE13507 datasets. Multivariate Cox analysis indicated that the IRG-based prognostic signature was independent of clinical factors. Further, it presented discrimination in overall survival rate, cancer-specific survival and progression-free survival in GSE13507 cohort, indicating the 7-IRG prognostic signature was independent of datasets. Using the 7-IRG signature along with age, gender and TNM_stage, we built a nomogram to predict overall survival for patients with BLCA. The nomogram promised to be clinically-relevant and credible in predictive performance. Additionally, we explored the underlying mechanisms using GSEA and immune cell analysis between risk groups. These findings support the potential translation of the present IRG-based prognostic signatures into clinical practice.

Our IRG-based signature highlighted seven IRGs, i.e., RBP7, PDGFRA, AHNAK, OAS1, RAC3, EDNRA, and SH3BP2. Expression of PDGFRA was reported in BLCA specimens; however, such report lacked sufficient experimental study (Terada, 2009; Terada, 2013). AHNAK was identified as a unique intracellular protein with different expression level and subcellular localization between BLCA sample and control (Lee et al., 2018); whereas, its prognostic value was observed in other studies (Chu et al., 2018; Wu et al., 2019). High expression of EDNRA was found to be associated with poor outcome in patients with advanced BLCA in a bioinformatics report (Laurberg et al., 2014). By contrast, no reports concerning RBP7, OAS1, RAC3, and SH3BP2 were published in BLCA, and therefore, the role of these IRGs in BLCA requires further investigation.

To explore the mechanisms by which the IRG-based signature effectively stratifies BLCA patients, GSEA between risk groups as classified by the signature demonstrated significant activity of multiple immune-related pathways in the high-risk patients. DEGs between risk groups were significantly enriched in chemokine signaling pathway and cytokine-receptor interaction, which are involved in chemotaxis, angiogenesis, as well as inflammatory processes (Lippitz, 2013; Li et al., 2017). The cytokines play a crucial role in the immune response of cytotoxic T lymphocytes (CTL), and the regulation of cellular differentiation (Agarwal et al., 2006). Besides, an enhanced inflammatory milieu is reported to be associated with tumor progression and poor prognosis (Lin and Karin, 2007; Lippitz, 2013). To further elucidate the role of tumor milieu associated with the 7-IRG signature, we analyzed the estimations of six types of immune cells and observed increased abundance of CD4+ T cells, CD8+ T cells, macrophage, neutrophil and dendritic cells in the high-risk group. T cells infiltration was reported to promote tumor invasion and metastasis *via* the androgen receptor (Tao et al., 2016) and estrogen receptor signaling (Tao et al., 2018) among BLCA patients. This aspect aligned with a clinical study that demonstrated the prognostic role of a specific subset of CD4+ T cells (Th17) (Chugh et al., 2013). The infiltration of tumor-associated macrophages is recognized to facilitate tumor progression in BLCA *via* tumorigenesis, angiogenesis, and disruption of adaptive immune response (Miyake et al., 2016). Moreover, the recruitment of neutrophils could increase the level of human neutrophil peptides, which cause tumor angiogenesis and growth (Thompson et al., 2015). An increase of tumor-infiltrating neutrophils was linked to immunosuppression in muscle invasion of BLCA patients that lead to inadequate treatment response and prognosis (Zhou et al., 2017). Additionally, the presence of tumor-infiltrated dendritic cells is associated with tumor progression and poor prognosis. By contrast, higher B cell level in the low-risk group indicated the tumor-suppressive role of B cell infiltration, which was consistent with a previous study (Jiang et al., 2019). In summary, the misregulation of tumor immune microenvironment may be responsible for the difference in survival outcome observed between groups as defined by the prognostic signature.

In the present study, we reported a prognostic signature based on IRG expression for predicting survival rate in BLCA patients, which was observed to be clinically relevant and effective in different datasets. To our best knowledge, this has been the first reported IRG-based signature in BLCA. Nevertheless, our results consisted of several limitations. First, as non-tumor samples were less than BLCA specimen, the results were biased to an extent. Second, the molecular mechanisms of BLCA could not be fully elucidated due to lack of *in vitro* and *in vivo* studies. Further studies are therefore warranted.

## Conclusions

We developed and validated a first-ever IRG-based prognostic signature that stratify BLCA patients into two subgroups with statistically different survival outcomes, for which misregulation of tumor immune microenvironment might be responsible. These findings may provide insight on development of novel immune biomarkers and target therapy.

## Data Availability Statement

Expression profile can be accessed in The Cancer Genome Atlas (TCGA) (https://portal.gdc.cancer.gov/) and gene expression omnibus (GEO) database (https://www.ncbi.nlm.nih.gov/geo/), while immune related gene list can be retrieved from the Immunology Database and Analysis Portal (IMMPORT) website (https://www.immport.org). Abundance of tumor-infiltrating immune cells were retrieved from the Tumor Immune Estimation Resource (TIMER) website (https://cistrome.shinyapps.io/timer/). The data sets used and/or analyzed during the current study are available from the corresponding author on reasonable request.

## Author Contributions

HQ, XH, CH, YL, and BY contributed to data analysis, interpretation, and drafting. HQ, XH, CH, YL, and JL contributed to study design, study supervision, and final approval of the manuscript. All authors read and approved the final manuscript.

## Funding

The study was supported by The Introduced Project of Suzhou Clinical Medical Expert Team (SZYJTD201725, recipient: JL) and Key Project of Jiangsu Provincial Department of Science and Technology (BE2017007-5, recipient: YL).

## Conflict of Interest

The authors declare that the research was conducted in the absence of any commercial or financial relationships that could be construed as a potential conflict of interest.

## Supplementary Material

Supplementary Table S1 Differentially expressed genes between BLCA samples and non-tumor samples.

Supplementary Figure S2 Boxplot of expression levels in these genes between BLCA and normal samples in TCGA dataset with results of Wilcoxon test.

Supplementary Figure S3 Riskplots for the 7-IRG signature in GSE13507 [(1)overall survival; (2) cancer-specific survival; (3) progression-free survival] **(A)** Heatmap of expression profiles of included IRGs **(B)** Distribution of groups based on the signature (C) Survival status of patients in different groups.
