# Supplementary figures and images for: Identification and Validation of an Individualized Prognostic Signature of Bladder Cancer Based on Seven Immune Related Genes
Source: Front Genet. 2020 Feb 5;11:12. doi: 10.3389/fgene.2020.00012 (PMC7013035; doi:10.3389/fgene.2020.00012)

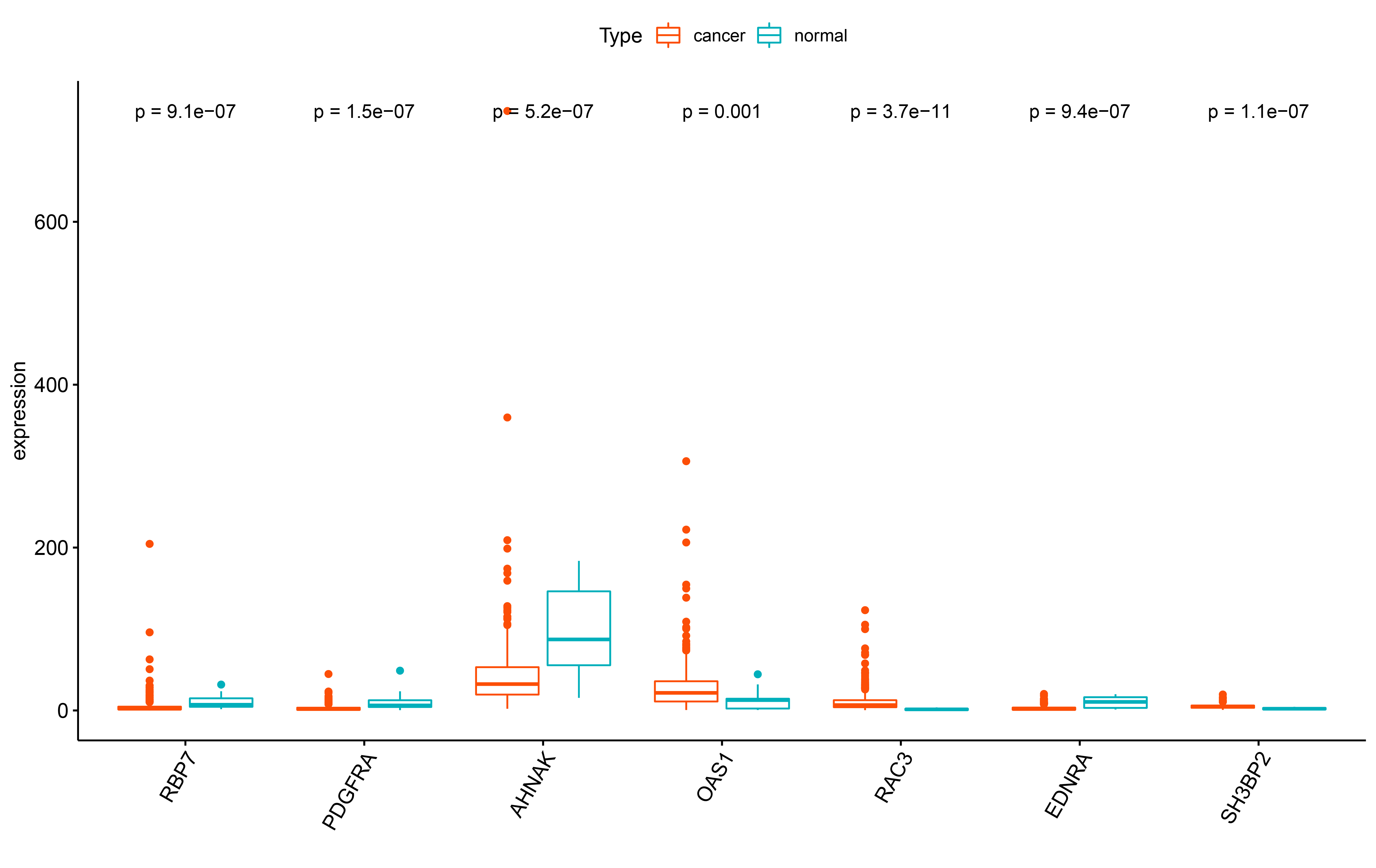

Supplement: Supplementary Figure S2 — Boxplot of expression levels in these genes between BLCA and normal samples in TCGA dataset with results of Wilcoxon test. [file Image_1.tif]

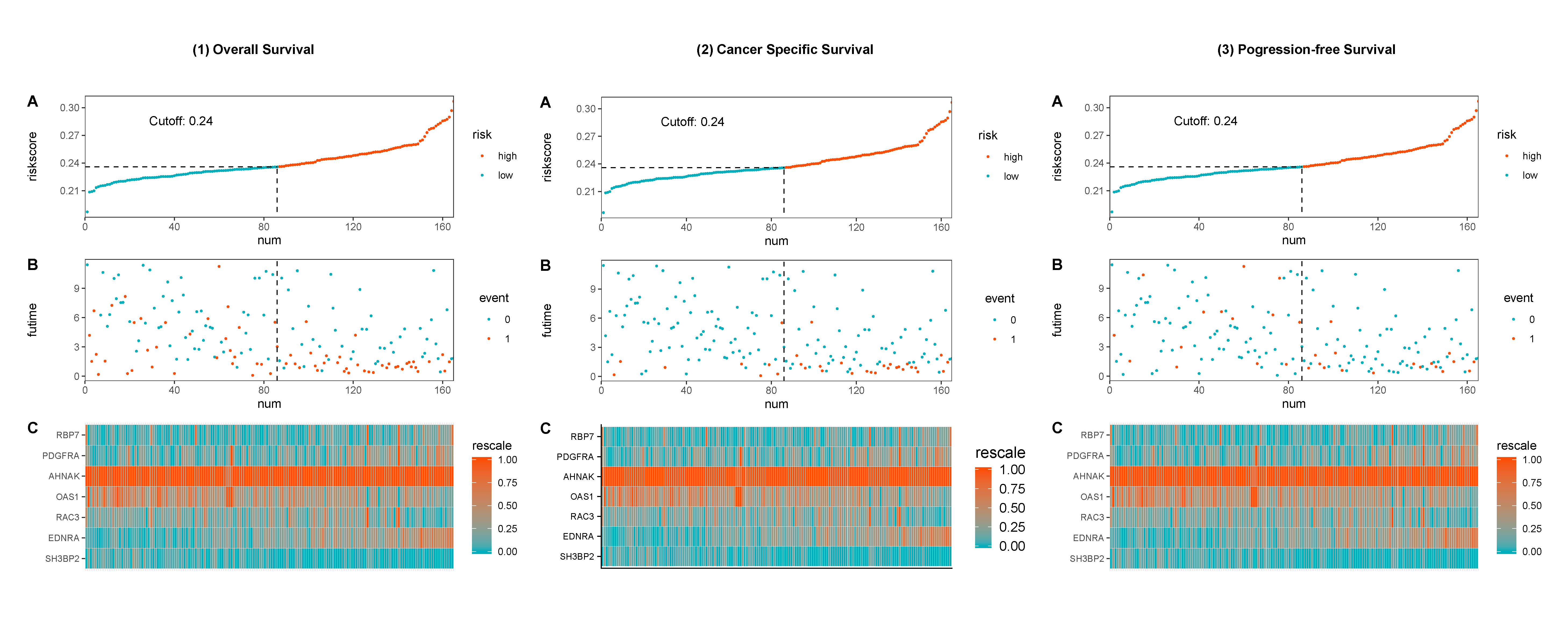

Supplement: Supplementary Figure S3 — Riskplots for the 7-IRG signature in GSE13507 [(1)overall survival; (2) cancer-specific survival; (3) progression-free survival] (A) Heatmap of expression profiles of included IRGs (B) Distribution of groups based on the signature (C) Survival status of patients in different groups. [file Image_2.tif]
